# Supplementary material for: A comparison of vertebral preparation techniques for increasing reader precision and agreement in vertebral band pair identification of Northeast Atlantic skates
Source: PLoS One. 2026 Feb 11;21(2):e0341911. doi: 10.1371/journal.pone.0341911 (PMC12893599; doi:10.1371/journal.pone.0341911)
Supplement: Attachment — Submitted filename: Response to Reviewers.docx [file pone.0341911.s003.docx]

Rebuttal Letter of the manuscript PONE-D-25-37758 entitled ‘Age determination in Raja brachyura, Raja clavata, and Raja montagui from the Northeast Atlantic: a comparison of different techniques’ submitted to the PLOS ONE.

Dear Joel Gayford,

We thank the reviewers for their positive comments concerning our manuscript. We have checked the general requirements of the journal as suggested and ensured the data are now publicly available via the DANS Data Station Life Sciences database repository.

Both reviewers highlighted the need for clearer contextualization of ageing methodologies, particularly the conventions of sectioning anterior vertebrae, and concerns were raised about the use of modal ages and the potential for underestimation. Reviewers also requested greater clarity and detail in the Methods and Results, including sampling effort, and statistical reporting. In response, we have revised the Introduction to better reflect best practices, added methodological and statistical detail, and reframed the Discussion to emphasize both precision and accuracy of age estimation. We also address the reviewers’ comments individually

Comments from the Editor

Journal Requirements:

When submitting your revision, we need you to address these additional requirements.

1.Please ensure that your manuscript meets PLOS ONE's style requirements, including those for file naming. The PLOS ONE style templates can be found at

<https://journals.plos.org/plosone/s/file?id=wjVg/PLOSOne_formatting_sample_main_body.pdf> and

[https://journals.plos.org/plosone/s/file?id=ba62/PLOSOne_formatting_sample_title_authors_affiliations.pdf](https://eur03.safelinks.protection.outlook.com/?url=https%3A%2F%2Ftrack.editorialmanager.com%2FCL0%2Fhttps%3A%252F%252Fjournals.plos.org%252Fplosone%252Fs%252Ffile%253Fid%3Dba62%252FPLOSOne_formatting_sample_title_authors_affiliations.pdf%2F1%2F010f01992813b2f9-2fc71487-6f14-4c6d-ba4f-3debf5e19216-000000%2FPKpadfag_g19qSK9qE9iRVHu6YuyV1DC6dL9npTcM18%3D227&data=05%7C02%7Celeanor.greenway%40wur.nl%7C24e72025f3e545a3ff9d08ddeea37a2b%7C27d137e5761f4dc1af88d26430abb18f%7C0%7C0%7C638929108585986829%7CUnknown%7CTWFpbGZsb3d8eyJFbXB0eU1hcGkiOnRydWUsIlYiOiIwLjAuMDAwMCIsIlAiOiJXaW4zMiIsIkFOIjoiTWFpbCIsIldUIjoyfQ%3D%3D%7C0%7C%7C%7C&sdata=GLi%2FfOmqcCmjoeuFn2x11TF%2F6Qb1i1zod9zEGRkug8s%3D&reserved=0)

The manuscript style requirements were double checked and changes were made to match the example document where necessary.

2. In your Methods section, please provide additional information regarding the permits you obtained for the work. Please ensure you have included the full name of the authority that approved the field site access and, if no permits were required, a brief statement explaining why.

These methods of sampling in this study did not involve any additional mortality outside of standard fishing practices and researchers had no involvement of killing fish used in this study. Therefore, this research did not require any animal experimentation permits. The following text has been added, line 121 – 127:
*“*Individuals of *R. brachyura*, *R. clavata*, and *R. montagui* were collected between June 2018 and March 2024. S*amples and data were collected either from landed individuals intended for human consumption, or as part of routine data collection in a commercial fisheries program for discard sampling in The Netherlands. This study was non-experimental as samples obtained from discard sampling involved opportunistically collecting individuals that were already deceased, where The Dutch Experiments on Animals Act does not apply. An ethical review by the Statement Animal Experiment Committee was therefore not required. No additional mortality or animal discomfort beyond standard fishing operations was caused for sample collection for the purpose of this study.”*

3. When completing the data availability statement of the submission form, you indicated that you will make your data available on acceptance. We strongly recommend all authors decide on a data sharing plan before acceptance, as the process can be lengthy and hold up publication timelines. Please note that, though access restrictions are acceptable now, your entire data will need to be made freely accessible if your manuscript is accepted for publication. This policy applies to all data except where public deposition would breach compliance with the protocol approved by your research ethics board. If you are unable to adhere to our open data policy, please kindly revise your statement to explain your reasoning and we will seek the editor's input on an exemption. Please be assured that, once you have provided your new statement, the assessment of your exemption will not hold up the peer review process.

The data were under review in the DANS data repository and are now publicly available with the doi link as stated in the submission process: <https://doi.org/10.17026/LS/ARNWEH>.

4. We note that Figure 1 in your submission contain map/satellite images which may be copyrighted. All PLOS content is published under the Creative Commons Attribution License (CC BY 4.0), which means that the manuscript, images, and Supporting Information files will be freely available online, and any third party is permitted to access, download, copy, distribute, and use these materials in any way, even commercially, with proper attribution. For these reasons, we cannot publish previously copyrighted maps or satellite images created using proprietary data, such as Google software (Google Maps, Street View, and Earth). For more information, see our copyright guidelines: <http://journals.plos.org/plosone/s/licenses-and-copyright>.

We require you to either (1) present written permission from the copyright holder to publish these figures specifically under the CC BY 4.0 license, or (2) remove the figures from your submission:

Figure 1 was created using the “rnaturalearth” package in R studio and is not a copyrighted image. This is now clarified in the figure legend with the following text “...*Locations were estimated from the national fisheries catch and effort database by linking fish landings with fishing trip locations, and visualised using the “rnaturalearth” package in R version 4.4.1.”*

Reviewer #1 synopsis:

The reviewer indicated that the manuscript would benefit from improved clarity, organization, and precision. They requested revisions for stronger phrasing, better flow, and clearer explanation of ageing structures and environmental influences. More detail on sampling effort, age-reading procedures, and statistical reporting, as well as clearer terminology and placement of sentences were also requested. The Discussion was viewed positively but would be strengthened by beginning with the study’s findings, restating results before interpretation, and refining certain statements for accuracy. In response, we revised the manuscript for clarity, reorganized sections, added methodological and statistical details, and refined interpretations in line with reviewer recommendations.

Reviewers' comments:
Reviewer #1:

1. L44 I think this sentence can be rewritten to be stronger ("fish" is used twice)

Changed as suggested

2. L50 give indication of why those structures are used in elasmobranchs (calcified, records age similar to otoliths); move parentheses to end of sentence ("sharks, skates and rays (elasmobranchs)"

Parentheses have been moved as suggested and the sentence has been changed to: ”*Alternative calcified structures, such as spines, rayfins, caudal thorns, and vertebrae, often feature patterns that reflect annual growth bands and are therefore used for age estimation in these species [6, 7].”*

3. L57-8 This sentence feels out of place here wince we don't have context for this particular species, but the inclusion of a specific study and species is something that should be done in the introduction.

We agree with the reviewer and the sentence has been adapted and moved towards the end of the introduction.

4. L59-61 More details on why the authors couldn't validate

We agree and now clarify why vertebral age validation can fail across elasmobranchs. Validation is often unachievable when growth bands exhibit inherently poor optical contrast and frequent accessory checks features that are species-specific and linked to vertebral cartilage biogeochemistry. In addition, band deposition can be non-annual or temporally variable among taxa and environments, and external validation approaches (e.g., oxytetracycline mark–recapture, known-age specimens, bomb-radiocarbon) are not universally applicable due to ethical, logistical, or life-history constraints.

5. L61 Unclear what the first discrepancy is, other than some papers invalidated vertebral growth

Sentence has been changed to “*Although age estimation in elasmobranchs has been validated in several studies [29-31], vertebral ageing cannot be universally confirmed because band visibility and periodicity vary among species; this discrepancy reflects interspecific differences in the biogeochemistry of vertebral cartilage, due to variation in matrix composition, degree of mineralization, and trace-element incorporation—factors influenced by diet and environmental conditions (e.g., temperature, salinity, dissolved oxygen, pH/carbonate chemistry, depth) [32, 33]. Moreover, the use of vertebrae for age estimation has been invalidated for several species, including the common sawshark (Pristiophorus cirratus) [34] and the Pacific angel shark (Squatina californica) [35]. As a result, a single standardized technique for age estimation across all elasmobranchs remains unlikely [6, 36]”*

6. L61-4 This sentence and first half of the second sentence can be combined

Changed as suggested.

7. L65 Provide examples of environmental factors (temperature? salinity?)

We now provide concrete examples of environmental drivers (temperature, salinity, dissolved oxygen, pH/carbonate chemistry, depth), with citations.

8. L77 More info on these "other studies" - species? underestimation? Etc

We added more details from the previous studies as suggested: “*However, other studies on different shark (Squatina dumeril, Carcharodon carcharias, Lamna nasus, Isurus oxyrinchus, Alopias vulpinus, Prionace glauca, and Carcharhinus obscurus) [41], skate, (Leucoraja erinacea, Leucoraja ocellata, Dipturus laevis), and ray species (Dasyatis sabina, and Urobatis halleri) [24] have reported age underestimations in posterior vertebrae as centrum morphology and band-pair counts vary along the vertebral column [24, 41]”*

9. L96-7 This sentence should be start of results

Sentence adapted to the results as suggested.

10. L109-111 I think this sentence is missing some punctuation

This sentence has now been broken into two sentences.

11. L111-12 Also put this in results

This particular section is currently in the methods section because it serves as support for our choice in vertebrae selection, which is detailed in the subsequent sentence.

12. L150 Eight readers at each institute or across all three?

This was eight readers across all three, sentence rephrased to: “*Age estimates were carried out independently by eight readers representing institutes in The Netherlands, Belgium, and Italy.”*

13. L223 "on average" a statistical average? Is it significant?

Average replace with ‘in most cases’.

14. Table 2 "Modal age range" is slightly confusing. I think it means the age range of the whole species sample as determined by the modal age of each individual across all reads, but it also could be the extent of the range in age read from the readers. Since it's already established that age was determined using mode read, "modal" can be removed from the term. Also, this needs units (years)

We agree with the reviewer and the word modal has been removed while including the unit (yr). We also added more information to the figure legend for clarity: “Table 2. Summary of ageing precision results of each vertebral preparation method. The age range in the precision measures column reflects the age range of the modal ages as estimated by all age readers.”

15. L234 "observed" implies the animal was monitored since birth and is thus validated to be 6. "estimated" would be more appropriate, since the true age is never known unless the animal was born and kept in captivity.

Changed as suggested.

16. Sections 3.2-3.3 These sections are missing reported test statistics (especially p values) and values of what is being compared..

The authors regret this information was missing form the original text, significant p-values and test statistics are now given in the text. Non-significant results are given in the figures.

17. L282 "each both" is redundant

Corrected as suggested.

18. L346-7 Start discussion with your findings; are elasmobranchs understudied? That wasn't mentioned in the introduction; I recommend a broader opening paragraph where a recommendation of best method is made, like last sentence of abstract and in the conclusion.

We thank the reviewer for their comment and the paragraph in the beginning of the discussion has been changed as suggested.

19. L370 "observed" -> "estimated"

Corrected as suggested

20. L371 The species doesn't live to 54, individuals in the species can live to 54

Corrected as suggested

21. L372 "a minimum of 54 years" sounds like all tope sharks must live to 54. Change to "can live to at least"

Corrected as suggested

22. L379 This sentence says the same thing... why would it say "although" if the age composition and growth curve are both similar in the two species?

The sentence has been adjusted to improve clarity: “S*tatistical significance of growth curves between anterior and posterior vertebrae was only detected for R. clavata (Table 3; Fig 6B). Although R. brachyura presented an age composition similar to R. clavata, the growth curves obtained for anterior and posterior vertebrae were similar (p = 0.35; Chen test)”*

23. L380 Give p value, not "no significant difference"

P-values were added as suggested.

24. L391 Sentence end?

Sentence corrected to ‘*while being more comparable to values from the Irish Sea’*

25. L394-5 This is a fairly large sample size for elasmobranchs, but not as much compared to teleosts

We added text to clarify that our sample size is particularly small for R. montagui, especially compared to teleost studies as the reviewer correctly identifies.

26. 399 Start section 4.2 with this study's findings on vertebral structure. The beginning of every section should start with restating the relevant findings from the results (lines 404-408)

Paragraph re-arranged as suggested.

27. 409 It's not expected, but it is common

Changed as suggested

28. L411 "APE and CV were lower, and in general PA was higher" This sentence also appears in line 222, and I'm confused by it again. It would help to give values with these types of statements such as APE and CV were x% lower and 75% of PA was higher than... In general, it's best to avoid saying "in general" (I couldn't resist):

Line 222 (now line 265) has been edited to include specific values derived from the table: “*Both APE was lower by 2–19% and CV by 3–25% for anterior, whole, stained vertebrae compared to sectioned vertebrae for all species. PA was also consistently higher by 13–14% for whole compared to sectioned vertebrae. In most cases, anterior vertebrae resulted in CV values 1–17% lover compared to posterior vertebrae, regardless of vertebral structure or staining (Table 2).*”

The discussion has also been adjusted to: “*Despite this, precision measures (APE, CV, and PA) indicated greater consistency and reliability for anterior, whole, stained compared to sectioned vertebrae for all species (Table 2).”*

In general, we agree with the reviewer that in general is it best to avoid ‘in general’.

29. 415 There's an extra "vertebrae" in this sentence

Deleted where necessary

30. L437 This is a really great and valuable section, but I would have liked to see it introduced in the introduction so that the reader has an understanding of why it is important to improve age studies in elasmobranchs

We thank the reviewer for their comment, we have now moved part of this section to the introduction.

31. Conclusion: Good, but maybe not necessary as it's own section. If the last sentence is removed, it can be added to the discussion. Up to the authors, but I don't think this text needs to be on its own.

We appreciate the constructive comment but we decide to keep the conclusion section separately.

32. L456 Change "the authors suggest" to "the findings suggest" or "the findings point to" so that the suggestion is driven by data, not the authors' interpretation

Changed as suggested.

33. L462-3 Make into one paragraph?

Changed as suggested.

34. L465-7 Unnecessary sentence

Deleted as suggested.

Reviewer #2 Synopsis

The reviewer indicated that the manuscript needs stronger contextualization of best practices in age estimation, particularly the convention of sectioning anterior vertebrae to minimize under-ageing. Concerns were also raised about the use of modal ages instead of consensus readings, which may bias results toward underestimation. They cautioned that the study may prioritize improving precision metrics over producing the most accurate or precautionary age estimates, and stressed that encouraging the use of whole vertebrae could be misleading for older individuals. In response to these issues addressed the line comments and reframed the manuscript align with established conventions and best practices.

Reviewer #2: General Comment to Authors

1. The introduction does not do a good job of situating the current ‘best practice’ of preparing and reading band pairs in vertebrae. The convention is to section anterior vertebrae as this generally results in higher age estimations; important for a field prone to systematic under aging through vertebral analysis, and convention since 2004 (Cailliet and Goldman 2004).

While we appreciate the comment from the reviewer, in our view, the convention to section vertebrae is not as strong as suggested for Raja species which have recently been aged with whole vertebrae (Thys et al., 2023; Bellodi et al., 2024) and not unequivocally supported by previous observations. We have adapted the introduction which emphasises the uncertainty in both vertebral preparation methods.

“*Comparisons of vertebral preparation methods have also shown variable results among elasmobranch species. Previous research shows that sectioned vertebrae produce higher and more accurate ages compared to whole vertebrae in as seen in the spot-tail shark (Carcharhinus sorrah), the Australian blacktip shark (Carcharhinus tilstoni) [36], and the Port Jackson shark (Heterodontus portusjacksoni) [37]. In contrast, other studies show that whole vertebrae produce similar ages to sectioned vertebrae in young blue shark (Prionace glauca) [38], or may even be preferred over sectioning vertebrae as in the crocodile shark (Pseudocarcharias kamoharai) [39]. Regardless, age underestimations occur in both whole and sectioned vertebrae in older individuals as growth bands either become tightly grouped on the vertebral edge making them more difficult to distinguish [40] or growth bands deposition halts once somatic growth slows or ceases [24, 41]. Such age underestimations have been validated from tagging studies [36, 42, 43], or bomb radiocarbon dating [44, 45]. Collectively, these studies highlight both the uncertainty and limitations of using vertebral centra for age estimations and demonstrate that preparation methods are highly species-specific. However, vertebral ageing appear most reliable for shorter-lived species < 20 years for which age estimates have been validated [36, 40, 42, 46].”*

2. It is not explained how the authors identified the birth mark between methods.

We regret this information was missing from the original text. The following text has now been added: *“Images were uploaded to ICES SmartDots software (v4.1) where ages were determined by counting pairs of opaque and translucent bands, starting with the first band pair after the birthmark. Birthmarks were easily identified in both whole and sectioned vertebrae as the first distinct opaque band distal to the focus [64] (Fig 2). In sectioned vertebrae, this is sometimes associated with an angle change in the corpus calcareum, although it is not always evident [6].”*

We have also updated Figure 2, which now include annotations of the band pair counts and the birthmark.

3. The use of taking the ‘modal age’ in lui of consensus reads among agers is prone to introducing systematic under ageing bias – what if the oldest age estimates were most true of actual age? That consideration cannot be made within the current methodology. The variation on age estimates (fig 4 & 5) is quite large and only emphasizes my concern. The present method selects for comfortable age-bias/precision stats, but does not necessarily ensure that the most true/best/pre cautionary final age estimations have been made in reality.

We agree with the reviewer that the use of modal age can potentially introduce a systematic under-ageing bias, particularly if the highest (oldest) age estimates more closely approximate true age. However, our primary objective was not to determine absolute accuracy but rather to assess how different preparation methods influence the readability and consistency of vertebral age estimates across multiple readers. The modal age approach enabled us to quantify reader consensus and exclude indeterminate cases (i.e., instances with no clear mode), thereby focusing the analysis on relative precision rather than absolute accuracy.

We acknowledge that this approach may overlook cases where the oldest age estimates, assigned by only one or two readers, might be closer to the true age, a limitation especially relevant for long-lived elasmobranchs, where age determination becomes increasingly uncertain with size and age. Although the species examined in our study are not among the most long-lived, we recognize that under-ageing is a well-documented issue in elasmobranch ageing and that this bias should be considered when interpreting our findings.

Nonetheless, the use of modal age remains a well-established and widely accepted practice in age and growth validation studies as a measure of ageing precision and reproducibility among readers (e.g., Campana 2001; Goldman 2005; Vitale et al., 2021; ICES, 2022). Given that our study aimed to evaluate the relative performance of vertebral preparation methods in improving reader agreement, rather than to establish definitive age estimates, we consider the use of modal age to be appropriate and consistent with established methodologies.

Our results, indicating that anterior, whole, unstained vertebrae yield the highest reader precision and agreement, should therefore be interpreted within this framework—acknowledging both the strengths of improved consistency and the inherent limitations associated with potential under-ageing bias. A brief sentence regarding this point has been added in the discussion.

4. The modeling approach of length at age data is quite antique. Its pretty firmly accepted to use a multi-model approach (not just a priori selection of the von bertalanffy function, Smart et al 2016)), and better yet a Bayesian model where priors of readily available information on size-at-birth/hatching and maximum size can be used to constrain the parameters of L0 and Linf (Smart and Grammar 2021).

We agree with the reviewer on the usefulness of applying a multi-model approach for studying elasmobranch growth, but we did not integrate this strategy in the present work simply because we did not consider it necessary for its ultimate scope. In fact, given the reduced number of observations (we only used those individuals for which both anterior and posterior vertebrae were available), growth modelling in this paper was conducted exclusively to test whether the two types of vertebrae provided the same results in terms of growth curves. For this reason, we decided to rely only on the von Bertalanffy function, as it is more easily comparable with the existing literature. Therefore, we deliberately avoided the use of multiple growth models (including Bayesian approaches), since the main purpose here was just to verify whether anterior and posterior vertebrae returned different growth patterns. Moreover, considering the small sample size, we were concerned that applying a multi-model comparison could introduce a potential bias by indicating one model as “better” without a solid basis, and at the same time would make the manuscript unnecessarily longer and more complex.

That said, we fully agree with the reviewer’s point: once the methodological framework is established in this study, our plan is to submit a second paper, based on a much larger dataset and a broader geographical scale, where we will conduct a proper multi-model (and possibly Bayesian) growth pattern analysis as suggested.

5. My final comment extending from 429 argues that this study has selected the best preparation method for vertebrae to improve age bias/precision metrics, not necessarily the method that would produce the most accurate estimation of true age. The concept of counting whole vs section vertebrae is again quite antiquated and really, its the bias in younger age classes in this study which is most likely what has resulted in comparable age estimates between whole and sectioned vertebrae. It’s a dangerous assertion to encourage use of whole vertebrae for skate on the back of this work, as it will not be appropriate for individuals attaining older ages than the present studies sample size. This is known from over 40 years of age and growth research, and goes against best practice guidelines of research in the field that has led to the presently accepted conventions on vertebrae preparation for ageing.

We thank the reviewer for their comment. Although the reviewer views this study as ‘antique’, ageing in Raja species is equally conducted with both whole (Thys et al., 2023; Bellodi et al., 2024) and sectioned (Carbonara et al., 2020; Bellodi et al., 2024) vertebrae as evidenced from studies over the past decade. This study provides the missing gap in knowledge to compare these two preparation methods for these three species. We agree that the mostly younger age classes were sampled in this study, but these are also shorter-lived species with a maximum estimated age of ~20 years, making sampling the older age classes more challenging (this has also now been included in the introduction). We also agree with the reviewer that encouraging the use of whole vertebrae is not suitable for all skate species, however here for the three species we have demonstrated that whole vertebrae either produce higher ages (*R. brachyrua*) or ages equal to that of sectioned vertebrae (*R. clavata* and *R. montagui*).

Line Comments
6. Line 51 Add caudal thorns to this list.

Added as suggested.

7. Line 57 Reframe this paragraph; staining is not always used, and to my awareness its probably more common not to stain. There are studies that have discussed the relative null effect of staining, and these should be referred to here. See for example Burke et al. 2020, which subsequently cites some useful papers exploring the use of staining; along with others cited in the present MS. The other key point to note with staining (more for discussion) is that this technique was popular before photographic manipulation was possible. These days, its probably more effective to play around with brightness and contrast of images to achieve the same effect as staining – this is why staining is hardly used now; digital advancements have made it largely superfluous. Along with this is the development of techniques like XRF elemental mapping, where calcium and phosphate (the most abundant elements) can be discreetly viewed along the transect.

-Burke, P. J., Raoult, V., Natanson, L. J., Murphy, T. D., Peddemors, V. & Williamson, J. E. (2020). Struggling with age: Common sawsharks (Pristiophorus cirratus) defy age determination using a range of traditional methods. Fisheries Research 231, 105706.

We thank the reviewer for their comment however we do not entirely agree. There are studies which did not used staining as the reviewer has pointed out (Drew et al., 2015; Pabon-Aldana et al., 2022; Meja et al., 2024; van Werven et al., 2025), but there are still multiple studies which have used and tested staining techniques in the past decade (Girgen and Basusta, 2016; James et al., 2024; David et al., 2025). These studies are species-specific and again highlights the need for the present study. We have now included this in the introduction and provided additional references taken from Burke et al (2020) in the introduction as suggested.

*“Despite the fact that elasmobranchs remain a largely understudied group, there is an abundance of different ageing techniques [4, 11] which have been used across multiple elasmobranch species. Currently, vertebrae used for ageing in elasmobranchs are either examined as whole structures or are sectioned. Staining is then often used to optimise the visibility of growth increments [12], where a range of staining methods have been tested [13] including alizarin red [4, 14, 15], silver nitrate [16, 17], cedar oil [4, 18], and crystal violet [17, 19-21]. Research over the past decade continues to refine or adapt these staining techniques [22-24]. However, staining is not always applied [25-27] and can sometimes be ineffective [6, 28], where recent studies make use of editing software to digitally enhance growth bands [27].”*

We have also addressed the use of new techniques in the discussion:
*“A variety of staining methods are often used on vertebral structures to improve clarity and readability of band pairs [85-89]. However, stains or dyes are not always applied and may not always enhance growth band visibility for age reading [6, 25, 27, 28] and tend to be species-specific [13, 61]. This study found that staining was not always successful. In some cases, traces of the staining solution remained on the vertebrae making it more difficult to differentiate band pairs. Despite this, staining had little effect (positive or negative) on age reading precision and readability for vertebrae regardless of vertebral location or structure for all three species (Table 1). Future studies may take advantage of emerging techniques such as using elemental analysis including laser ablation inductively coupled plasma mass spectrometry [27, 90-92] and near-infrared spectroscopy [93, 94] to corroborate age readings from vertebrae.”*

8. I also think it needs to be clear that vertebrae are most commonly sectioned, as previous work has identified under aging of whole centra;
Discussed a bit in following + other papers already cited in this MS. This introduction doesn’t really set the appropriate context for accepted methodological conventions in the field that are designed to improve readability and minimise under aging. This is important context to this present work – the convention is to section anterior vertebrae; this study largely seeks to validate or invalidate this convention for these species of skate.

Harry, A. V. (2018). Evidence for systemic age underestimation in shark and ray ageing studies. Fish and Fisheries 19, 185-200.
Cortés, E., Grant, M. I., Dureuil, M. & Ellis, J. R. (2024). Life history characteristics. In: R.W. Jabado, A.Z.A. Morata, R. Bennett, B. Finucci, J. Ellis, S. Fowler, M.I. Grant, A.P.B. Martins and S. Sinclair (Eds.), The Global Status of Sharks, Rays, and Chimaeras, pp. chapter 3, 33–38. IUCN/SSC Shark Specialist Group. IUCN, Gland, Switzerland and Cambridge, UK
Goldman, K. J., Cailliet, G. M., Andrews, A. H. & Natanson, L. J. (2012). Assessing the age and growth of chondrichthyan fishes. In Biology of Sharks and Their Relatives (Carrier JC, Musick JA & MR, H., eds.), p. 423. Boca Raton, FL: Taylor & Francis Group.
MacNeil, M. & Campana, S. (2002). Comparison of whole and sectioned vertebrae for determining the age of young blue shark (Prionace glauca). Journal of Northwest Atlantic Fishery Science 30, 77-82.

As addressed in the previous comment, we have added more information to the introduction highlighting that both whole and sectioned vertebrae are used for ageing *Raja* species and uncertainty of ageing using these methods.

9. Line 64 The biochemical structure of vertebrae (phosphate hydroxyapatite [Ca10(PO4)6(OH)2]) is not variable between species. Its density, flexibility are influenced most likely by species morphology and stress forces needed in skeletal structure owing to swimming mode and ecological activity (along the spectrum of sedentary to highly mobile- if a species is swimming a lot, it needs a string skeleton to anchor large muscles to). Here, diet and other environmental factors are referred to, This releates to trace element incorporation in the Ca10(PO4)6(OH)2 matrix or elements entrapped in the protein matrix. These factors would only have minor relevance to structural properties (density, hardness, etc) of vertebrae in the context the authors are talking about here. These needs some revision to better describe structural properties of vertebrae, there composition, and what factors influence composition. Lisa Natanson explored these concepts late in her career;

James, K. C. & Natanson, L. J. (2020). Positional and ontogenetic variation in vertebral centra morphology in five batoid species. Marine and Freshwater Research 72, 887-898.
Natanson, L. J., Skomal, G. B., Hoffmann, S. L., Porter, M. E., Goldman, K. J. & Serra, D. (2018). Age and growth of sharks: do vertebral band pairs record age? Marine and Freshwater Research 69, 1440-1452.
Natanson, L., Andrews, A., Passerotti, M. & Wintner, S. (2018). History and Mystery of Age and Growth Studies in Elasmobranchs: Common Methods and Room for Improvement. In Carrier, J. C., Heithaus, M. R., & Simpfendorfer, C. A. (Eds.). Shark research : Emerging technologies and applications for the field and laboratory, pp. 177-194. Taylor and Francis Group. pp. 178-194.

However, I wonder how relevant this really is to the present paper? It hasn’t explored how varied vertebral structures hold up against vertebral preparation techniques. Better to set the context of conventional methods for vertebral preparation for ageing, and why it is we have arrived at the present convention of sectioning anterior vertebrae, where staining has limited benefit. That’s the state of knowledge this MS needs to build a hypothesis of that would improve the field.

We thank the reviewer for their comment and agree that this section needed some revision. We corrected the text to ‘biogeochemical’ structure and although this was not explored in the current paper, we feel this brief section helps emphasise that ageing in elasmobranchs in inherently difficult and that the success of ageing (and preparation methods) are species specific.

We have also included more information regarding the preparation methods and the use of staining in the context of more recent literature.

10. Line 154 Please explain the process of identifying the birthmark for whole and sectioned vertebrae. Its typical to get a translucent band at the birthmark, so did counts begin from the second translucent band in whole vertebrae? Another advantage to sectioned vertebrae is that the birth mark is more easily identified, as in addition to a thin translucent band that is typically (but not always) laid, is that there will usually be an acute angle change in the intermedalia at the birth mark, and sometimes a visible ‘notch’. These characters cannot be identified in whole vertebrae, so how was the birth mark identified? Was the accuracy of its identification corroborated with sectioned vertebrae? One of the major error sources of age estimation is mis identification of the birth mark, and this study has not explained how it was identified, and what constituted a growth band there after. It just says growth bands were counted.

This has been addressed in a previous comment and figure 2 has been updated to show birthmark and annual ring identification.

11. Line 187 It’s a bit of an old fashioned parametrization of the growth model used here. The parametrization that includes L0 is more useful, as L0 serves as a proxy for size at birth, with t0 has no biological meaning. The use of the parametrization with L0 also allows Bayesian A&G models to be used, and L0 and Linf can more easly have informed priors, based on knowledge of birth size and maximum size. Its probably not possible to accurate inform a prior of t0. Its not essential, but I would be highly encouraging of considering use of a more contemporary approach to modelling the length at age data. Please see
Smart, J. J., Chin, A., Tobin, A. J. & Simpfendorfer, C. A. (2016). Multimodel approaches in shark and ray growth studies: strengths, weaknesses and the future. Fish and Fisheries 17, 955-971.
Smart, J. J. & Grammer, G. L. (2021). Modernising fish and shark growth curves with Bayesian length-at-age models. PLoS One 16, e0246734. (includes very intuitive R package)
This section also needs to be clear that the a priori selected model is the von bertalanffy growth function. Again, see Smart et al 2016 above, as this strays from contemporary approaches to modelling length at age data whereby a multimodel approach is encouraged as best practice.

We do agree, please see the comment above.

12. Line 429 and then general discussion remarks
Yes, but only for the young spectrum of age estimates encountered in this study. This discussion is crying out for explicit mention that a key constraint here is the young ages encountered <8. The key advantage of sectioning comes when highly compressed ‘older age bands’ need to be examined, when animals have 20, 30, 40 band pairs to count. It is not really all that surprising that whole vertebrae performed equal, or slightly better than section vertebrae I some instances for these young age classes where growth bands are inherently larger and more easy to count – the need for sectioning was not born out of difficulties counting bands in younger age classes, it was from difficulties encountered in counting bands in older age classes, which are not included in this study.

Further, is there any insight to offer in where the larger age discrepancies in sectioned vertebrae arose? Was it birth mark, distal edge? That would be useful information; identifying vertebral zones leading to mis counts among readers.

The other factor here, is the assumption is made that band pairs equate to annual growth increments. There was more variability in the sectioned vertebrae, but how do you know that those age estimates were not better than whole vertebrae?

By not sectioning, smaller more indiscreet bands are hard to identify, and may have been missed. Our built ‘best practice’ of ageing calls for sectioning for this reason (e.g., Cailliet and Goldmans catelouge of papers on this topic; built from over 30 years of examining ageing studies and trailing methods; along with prior mentioned better ability to identify the birth mark; Cailliet and Goldman 2004). What this study has done is identify the best preparation method of vertebrae from these species of skate, in these ‘age’ classes, that allows a wide range of readers to most commonly agree on their ‘age estimates’ and thereby produce the best age bias/precision stats. Whether underaging has occurred is an un answered question. An interesting thing here, and counter to most literature, is that higher age estimates were made for R. brachyura with whole vertebrae, but not for the other two species. Why do you think this is? Have whole vertebrae resulted in better age-bias stats because ambiguous bands, or smaller bands are less clear in whole vertebrae compared to section vertebrae so that there is effectively less source for error ? – have whole vertebrae potentially missed some smaller bands that could well represent annual depositions under the assumption band pairs are accreted annually for these species? If there are less banding structures to count, age-bias stats are inherently going to be lower

We agree that the study is limited to younger age classes, but this is expected as the three *Raja* species of concern are shorter-lived species reaching a maximum age ~20 years (this has also now been included in the introduction), also determined with the use of sectioned vertebrae (Kadri et al., 2014; Carbonara et al., 2020; Bellodi et al., 2024).

We agree with the reviewer that discrepancies in the age readings would be useful information. Unfortunately, due to limitations in the age reading software, length of the growth increments were not recorded and could not be analysed further in this study.

We have addressed the reviewers comment regarding the use of whole and sectioned vertebrae in previous comments. We have also added a description of birth bands in the methods and added marks of the birth bands in figure 2 (as requested by the reviewer), where the birth marks are easily distinguished in these *Raja* species in both whole and sectioned vertebrae.

13. Line 237 states that sectioned vertebrae had higher ‘modal’ age estimates for R. clavate and R. montagui, but, these precision and bias stats were higher. Given that aging is about producing estimates that most reflect true age of the animal so that those ages can be fed into management initiatives, is it better to have higher more precautious ages with slightly worse age bias/precision stats, or lower more conservative ages with neater age bias/precision stats? Its an partly uncomfortable question that this study has raised, and it needs to be addressed. The goal of aging is not low APEs, its proving the best advice to management of growth of the species, and given we know underestimation occurs from vertebral ageing, it seems to make sense to use the precautionary principle and err toward older estimates, even if it means lower agreement among readers. Better to over age than under age.

Furthermore, the concept of selecting the ‘modal age’ is not something I entirely agree with – how to you know that the oldest age estimate wasn’t the more accurate to true age and that the modal age selection is not a form of methodological bias leading to systematic age underestimation? The variation on age estimates (fig 4 & 5) is quite large and only emphasizes my concern. Where any consensus reads done? (if not, again straying from conventional methodology), particularly with the ager who made the oldest age estimation for each individual so that they could make their case as to their decision to count the band structures they could identify? In a field where underage estimation is an issue, again, it’s a backward facing decision in respect to the history of this problematic field. The present method selects for comfortable age-bias/precision stats, but does not necessarily ensure that the most true/best/pre cautionary final age estimations have been made in reality. As such, the title is not entirely appropriate; it should be ‘Vertebral preparation to improve band pair identification agreement among readers’ or something to that effect.

These technicalities need some discussion. The field of ageing with vertebrae is plagued with inferences, and sometimes proven examples of systematic underestimation of age (e.g., the MRC study referred to for Tope, theres others for white sharks, where if we believed whole vertebrae estimates they’d have a max age of about 20, not 70+). My over arching point is that whole vertebrae may have resulted in clean APE’s and CV’s, but have they resulted in more accurate estimations of the age of these individuals – this is a separate point to the number of readers that agreed on an age, as that will err toward conservative counts. This discussion needs to consider these aspects – do whole vertebrae improve age estimates or just age precision metrics among readers? My view is that the latter is probably true and this discussion needs to be framed to answering this question as it’s the over arching unknown to come out of this study.

We thank the reviewer for their comment but we see that the reviewer has misinterpreted the text in this case. Our results show that sectioned vertebrae produce similar ages to those from whole vertebrae, where it was whole vertebrae that produced higher ages for only one species. Our results did indeed show that precision and bias stats were higher in sectioned vertebrae. Given that age estimates were similar between whole and sectioned, we advise using the method with the lowest age bias/precision values for these three species.

We have emphasised in the introduction that sectioning vertebrae also has its limitations as also shown in the literature (Harry et al., 2018; Natanson et al., 2018; James et al., 2021). We would also like to clarify that the MRC for Tope, estimated maximum age using sectioned vertebrae at ~20 years, whole vertebrae estimated maximum age at ~14 years (Moulten et al 1992; now added to the discussion), even though sectioned vertebrae produced higher ages (which we do not contest in this study) it emphasises the limitations which are also observed with using sectioned vertebrae for longer-lived species. We do not refute that sectioned vertebrae may result in higher ages in other longer-lived species, but rather show that for these three species age estimates from both preparation methods are similar - also seen in the blue shark (MacNiel 2002).

14. Some good well presented and discussed points are that anterior vertebrae resulted in higher age estimates generally, and were ‘more readable’ and that staining had very limited effect. This is corroborated in the literature, and this study adds to the evidence base of these factors. I have no issues with the PCA parts of this study, though I wonder if there is really enough latitudinal and longitudinal scope to provide robust insight. I’d also be interested to see the effects of reader experience on ages.

We thank the reviewer for their comment. The PCA results (Tables 4:7) showed that reader experience explained little of the total variance, this is addressed specifically in the text (lines 385-387). We also added more details regarding the categorisation of reader experience (line 239-243).

“*Age readers were categorised into experience levels based on the total number of vertebral readings where ‘low’ was less than 2000 vertebral readings, ‘medium’ was between 2000-5000 vertebral readings and ‘high’ was more than 5000 vertebral readings. Of the age readers in this study, one had ‘low’ experience, and one had ‘high’ experience, all others were ‘medium’ experienced*”.

15. Figure 2, please mark what was identified as the birth mark on this figure, and it would be useful to mark the identified band pairs counted also (if possible considering the modal age method). These images lack meaning otherwise for the reader

Added as suggested, see previous comment.
