# Supplementary material for: A comparison of vertebral preparation techniques for increasing reader precision and agreement in vertebral band pair identification of Northeast Atlantic skates
Source: PLoS One. 2026 Feb 11;21(2):e0341911. doi: 10.1371/journal.pone.0341911 (PMC12893599; doi:10.1371/journal.pone.0341911)
Supplement: Attachment — Submitted filename: Response_to_reviewers_auresp_2.docx [file pone.0341911.s004.docx]

Rebuttal Letter of the manuscript PONE-D-25-37758 with the updated title ‘*A comparison of vertebral preparation techniques for increasing reader precision and agreement in vertebral band pair identification of Northeast Atlantic skates’* submitted to the PLOS ONE.

Dear Joel Gayford,

We thank the reviewers for their final comments concerning the manuscript. We value their feedback and the manuscript has improved considerably from their input. We also updated our sample sizes where minor updates to the tables were also incorporated.

Reviewer 2 synopsis:
The reviewer recommends strengthening this justification further by explicitly embedding the rationale for using modal age within the Methods section, as it is a foundational methodological decision influencing interpretation of results. They also agree that restricting analyses to the von Bertalanffy growth function may be acceptable given the stated objectives, but stresses that this should also be clearly stated in the methods.

Reviewer #2: I thank the authors for their response to my comments and improvements made to the manuscript. At heart, I do not agree with the modal age approach, but I am satisfied that the first paragraph of the discussion outlines the key constraint for the reader, and the purpose of its use herein. There is scope to justify the reasoning for this approach in the methods additionally, as it is a foundational decision made in the conduct of the methodology of this study, and an action that influences the results and their interpretation (see below comment for a suggestion, as you’ve actually provided a really good couple of sentences in a reply to a comment).

1. Considering this text now added to the start of the discussion, and reply to my comment 3., I wonder whether the title is entirely appropriate. The final sentence of the first discussion paragraph for example is correct, and also implies the title is misleading
“Therefore, our finding that anterior, whole, unstained vertebrae provided the highest reader precision and agreement should be interpreted within this context”

A more appropriate title as such would be a synonym of:
A comparison of vertebral preparation techniques to increase reader precision and agreement in vertebral band pair identification in northwest Atlantic skates.

This is what the study has done. I recommend saving the ‘age estimation’ for the follow up manuscript that you mention

A few responses to previous comment reply’s and just a few more line comments below. Well done to the authors, this was quite a large review and I appreciate the time in constructing considered responses.

We agree with the reviewer and have updated the title as suggested.

2. Reply to comment 3.
The statement ‘modal age remains a well-established and widely accepted practice in age and growth validation studies’ is something I just do not consider to be true. Noting the authors list of referred to papers (Campana 2001; Goldman 2005; Vitale et al., 2021; ICES, 2022) in their response to comment 3., Campana 2001 for example speaks to ‘modal progression’ which is in the context of length class modes corresponding to ages, and as such, whether length modes in an annual breeding population can be used to estimate age from length; not taking the mode of age estimates across a range of readers who have examined banding patterns in calcified hard parts to determine age. I haven’t had much luck locating others cited, but ensure there is no confusion in ‘modal progression’ which is different to the present method. Regardless, better to justify the use of using modal ages to the readership more so than me. I like the justification provided in the first paragraph in the authors reply to the original comment 3., and would encourage its inclusion within the paragraph ending at line 194

‘…. modal age can potentially introduce a systematic underageing bias, particularly if the highest (oldest) age estimates more closely approximate true age. However, our primary objective was not to determine absolute accuracy but rather to assess how different preparation methods influence the readability and consistency of vertebral age estimates across multiple readers. The modal age approach enabled us to quantify reader consensus and exclude indeterminate cases (i.e., instances with no clear mode), thereby focusing the analysis on relative precision rather than absolute accuracy.’

We thank the reviewer for their comment and we have included the text in the manuscript as suggested.

3. Reply to comment 4.
This part of your response should be clearly stated in the methods speaking about which model was used
‘growth modelling in this paper was conducted exclusively to test whether the two types of
vertebrae provided the same results in terms of growth curves. For this reason, we decided to rely
only on the von Bertalanffy function, as it is more easily comparable with the existing literature.’

We agree with the reviewer and the sentence has also been included in the methods (now line 229-231).

4. Its good to hear a robust age and growth study on the way. A point of caution is that the below part of the response is fundamentally incorrect; its all about fitting the best model to the length-at-age data at hand to produce the best fit for your data. Not exploring the best model for the data used in caution of setting a precedent does not make sense, and I don’t understand what is meant. Perhaps you are referring to the underlying sample size considering the follow up paper you mention. In anycase, the intention of a multimodel approach is not to one day revert back to a priori selection of a single model, just want to make sure that is clear.
“Moreover, considering the small sample size, we were concerned that applying a multi-model comparison could introduce a potential bias by indicating one model as “better” without a solid basis,”

This response has not addressed the use of t0 – a biologically meaningless parameter that can very easily be recalculated to L0 from Linf and K:
L0 = L∞(1− exp(k*t0))

We understand the concern from the reviewer regarding t0 calculation. We have revised the Methods section to explicitly state that *t₀* is retained solely as a mathematical curve-fitting parameter and is not interpreted biologically. The relevant text has been added to the growth modelling section:
“*Curve fitting and growth model reliability near age 0, age–at–length data from eight R. clavata and ten R. montagui hatchlings born in captivity in the aquatic research facility of Wageningen University CARUS were added to the dataset. These observations were included used to constrain the early proportion of the growth curve, with the parameter t0 treated as a mathematical curve-fitting parameter.”* (line 232-236)

5. Line 40
Would be good to add the age range here, as per previous review these results may differ for older animals “…were more precise for age classes … , offering..”

We agree with the reviewer and the sentence has been changed to “…*were more precise for age classes 0 - 9 years, offering…”*

6. Line 117
This sentence is incomplete. State ‘draw conclusion on the most effective vertebrae preparation methods for the Raja genus’ or something similar.

The sentence has been changed to “…*we aim to draw conclusions on the most effective vertebral preparation techniques for the Raja genus*”.

7. Line 240-44
I wonder if a measure of the number of published age and growth papers each reader has been an author on, an/or led or been senior author on, and/or been an ager in, and/or number of sp. aged would be better metrics? I for example in my first age and growth paper read about 1500 vertebrae of a single species in two populations before proceeding with age estimates for that paper (n>500). That means under the present definition I would have medium experience despite at that point, only having aged one species and not having had an accepted publication yet; I’d consider my experience low at that point. Something to consider. I understand the present system captures the spectrum of present experience, but it doesn’t confer much to actual robust contribution to the field as a measure of expertise.

We thank the Reviewer for raising an important and conceptually relevant point regarding the definition and quantification of experience. We acknowledge that the approach adopted has limitations and does not aim to fully capture all dimensions of expertise in age and growth studies. Nevertheless, we believe that, despite its imperfections, it represents a pragmatic and easily implementable solution that is not necessarily inappropriate for the objectives of the present study.
The use of alternative metrics, such as the number of published papers or the number of species included in previous studies, would not necessarily provide a more accurate representation of actual experience. For instance, an individual may be listed as an author on age and growth studies involving multiple species without having been directly involved in age estimation or in the interpretation of growth patterns, having instead contributed through sample provision or analyses conducted after age determination. In such cases, experience based on publication metrics would likely be overestimated.

Similarly, while experience gained across different species may ensure greater familiarity with the general ageing process, it may also introduce potential bias by unconsciously interpreting the growth patterns of a new species based on prior experience that is not necessarily transferable or appropriate in the new biological context. In light of these considerations, we consider the adopted criterion, while admittedly imperfect, to represent a reasonable compromise between simplicity, transparency, and internal consistency, and to avoid some of the potential distortions associated with more complex but not necessarily more informative metrics.

8. Line 443
‘k’ in VBGF is not a growth rate but a coefficient of the curvature of the function that describes a proxy for the ‘growth completion rate’ determined from the curveature between L0 and Linf. Easiest to replace ‘growth rate’ with ‘k’ than to describe the nuances of this. It can be paired with statements like ‘higher k values indicates faster growth’. There are equations for calculating growth rate from VBGF parameters if wanting to pursue that, but I don’t see it as needed. It’s a common mistake in age and growth literature. In 4.1 discussion, and elsewhere please adjust use of 'growth rate' as a direct infernece from k, or substitute of using k.

Instances of ‘growth rate’ were replaced with ‘growth coefficient’.
